# The chromatin remodeling factors EP300 and TRRAP are novel SMYD3 interactors involved in the emerging ‘nonmutational epigenetic reprogramming’ cancer hallmark

**DOI:** 10.1016/j.csbj.2023.10.015

**Published:** 2023-10-12

**Authors:** Candida Fasano, Martina Lepore Signorile, Elisabetta Di Nicola, Antonino Pantaleo, Giovanna Forte, Katia De Marco, Paola Sanese, Vittoria Disciglio, Valentina Grossi, Cristiano Simone

**Affiliations:** a Medical Genetics, National Institute of Gastroenterology - IRCCS “Saverio de Bellis” Research Hospital, Castellana Grotte, 70013 Bari, Italy; b Medical Genetics, Department of Precision and Regenerative Medicine and Jonic Area (DiMePRe-J), University of Bari Aldo Moro, 70124 Bari, Italy

**Keywords:** Gastrointestinal cancer cell lines, Hallmarks of cancer, 'nonmutational epigenetic reprogramming' cancer hallmark, In silico tripeptide screening, SMYD3, SMYD3 interactome

## Abstract

SMDY3 is a histone-lysine N-methyltransferase involved in several oncogenic processes and is believed to play a major role in various cancer hallmarks. Recently, we identified ATM, BRCA2, CHK2, MTOR, BLM, MET, AMPK, and p130 as direct SMYD3 interactors by taking advantage of a library of rare tripeptides, which we first tested for their *in vitro* binding affinity to SMYD3 and then used as *in silico* probes to systematically search the human proteome. Here, we used this innovative approach to identify further SMYD3-interacting proteins involved in crucial cancer pathways and found that the chromatin remodeling factors EP300 and TRRAP interact directly with SMYD3, thus linking SMYD3 to the emerging ‘nonmutational epigenetic reprogramming’ cancer hallmark. Of note, we validated these interactions in gastrointestinal cancer cell lines, including HCT-116 cells, which harbor a C-terminal truncating mutation in EP300, suggesting that EP300 binds to SMYD3 via its N-terminal region. While additional studies are required to ascertain the functional mechanisms underlying these interactions and their significance, the identification of two novel SMYD3 interactors involved in epigenetic cancer hallmark pathways adds important pieces to the puzzle of how SMYD3 exerts its oncogenic role.

## 1 Introduction

The histone-lysine N-methyltransferase SMYD3 has been found overexpressed in several human cancers and has been linked to multiple cancer-related mechanisms, such as cell cycle deregulation, uncontrolled cell proliferation, migration and invasion, deregulated chromatin remodeling, altered gene expression, and histone and non-histone protein methylation [1].

SMYD3 is a member of the SET and MYND Domain (SMYD) lysine methyltransferase family, which includes multi-domain SET methyltransferases implicated in human cancerogenesis, cancer progression, and invasion [2]. The SMYD family consists of five members (SMYD1–5) that share two highly conserved domains: i) the MYND-type zinc finger domain, which is so called after the three best-characterized proteins containing it (MYeloid translocation protein 8 (MTG8/ETO), Nervy, and Deaf-1) and mediates protein-protein interactions, and ii) the SET domain, which contains the catalytic site responsible for lysine methylation [3]. While SMYD3 was first identified as an H3K4 di- and trimethyltransferase, subsequent studies revealed that it can also mediate the methylation of histone H4 at K20 and K5 [4]. Through its histone methylation activity, SMYD3 regulates the transcription of various oncogenes involved in liver and colon carcinogenesis, including MYC and CTNB1, as well as components of the IL6-JAK-STAT3 cascade [5]. SMYD3 can also interact with and methylate non-histone proteins. These methylation marks transactivate specific pathways involved in cancer cell survival and tumor progression [2]. In particular, SMYD3 has been shown to directly methylate VEGFR1 [6], MAP3K2 [7], and AKT1 [8].

Recently, we identified SMYD3 as an important effector of ten cancer hallmarks based on its direct interaction with ATM, BRCA2, CHK2, MTOR, BLM, MET, AMPK, and p130 [9], [10]. These findings suggest that SMYD3 may be involved in epigenetic reprogramming as part of its role in the multistep process of carcinogenesis [1]. Indeed, while epigenetic regulation of gene expression is a well-known mechanism governing embryonic development, differentiation, and organogenesis, nonmutational epigenetic reprogramming emerged as an enabling characteristic contributing to the acquisition of hallmark capabilities throughout cancer formation and progression and has thus been proposed as an additional feature to be included among the hallmarks of cancer [11].

Overall, a growing body of evidence indicates that SMYD3 is an essential epigenetic regulator that methylates histone and non-histone substrates, orchestrating protein-protein and protein-DNA interactions; however, its epigenetic role in cancer is not fully understood yet. In this report, taking advantage of an innovative *in silico* methodology, we identified TRansformation/tRanscription domain-Associated Protein (TRRAP) and E1A-associated Protein p300 (EP300, also known as P300) as novel SMYD3 interactors involved in the emerging ‘nonmutational epigenetic reprogramming’ hallmark.

TRRAP is a massive protein consisting of 3859 amino acids with a molecular weight of 434 kD, which, from a phylogenetic and functional standpoint, can be considered an ancestral member of the Phosphatidylinositol 3-Kinase-related Kinase (PIKK) family [12]. The C-terminal region of TRRAP contains a FAT and a PI3K/PI4K catalytic domain that are not found in its orthologs and paralogs [12]. TRRAP is a well-known component of numerous histone acetyltransferase (HAT) complexes. In particular, it orchestrates the assembly of these complexes onto chromatin during crucial processes such as DNA transcription, replication, and repair, and also coordinates other chromatin-related cellular functions [13]. TRRAP is a component of the STAGA and NuA4 HAT complexes, which comprise the acetylated nucleosomal histones H4 and H2A and are required for TP53, E2F1, and E2F4-mediated transcriptional activation [13]. In addition, TRRAP regulates transcription by linking transcription factors such as E1A, MYC, or E2F1 [13] and has been shown to modulate the activity of various cancer-related proteins, including MYC [14], TP53, MDM2 [15], and NPAT [16]. Therefore, TRRAP deregulation can potentially contribute to cancer development [13].

EP300 is a HAT and transcriptional coactivator with a multidomain structure comprising a catalytic core, multiple interaction domains, and various inter-domain intrinsically disordered regions (IDRs). The catalytic core contains a bromodomain, a RING-PHD domain (also known as CH2), and a HAT domain [17]. The bromodomain is essential for the recognition of lysine residues during substrate acetylation, and its absence negatively affects EP300 substrate specificity and transcriptional activity [17]. The PHD domain in the CH2 region is interrupted by a RING domain, resulting in a discontinuous structure. This RING domain has been found to exert an inhibitory effect on HAT activity [17]. EP300 acetylates all four core histones in nucleosomes, thereby regulating the transcription of several genes via chromatin remodeling, including ATF2, BCL6, and TP53 [17], [18], [19], [20]. For example, EP300 affects BCL6 expression through acetylation of histone H3 at lysine 122 (H3K122ac) and lysine 27 (H3K27ac) [18], [19], [20]. In addition, EP300 has also been shown to acetylate non-histone proteins, such as ALX1 [21], HDAC1 [22], and SIRT2 [23], and is part of multiple TRRAP-containing complexes, such as HAT complexes that regulate E1A and c-MYC activity and expression [24], [25].

Through its HAT activity, EP300 provides epigenetic tags for transcriptional activation and regulates important cellular processes, including cell proliferation, differentiation, and apoptosis, and its dysregulation has been associated with oncogenesis and cancer progression [26], [27], [28], [29], [30], [31].

This evidence highlights the crucial role played by SMYD3, TRRAP, and EP300 in cancer-related epigenetic reprogramming processes.

## 2 Materials and methods

### 2.1 *In silico* screening of P1-P19 tripeptides

The screening for rare tripeptides was performed as previously described [9], [10]. Briefly, a library of rare tripeptides (termed P1-P19) was set up, and each tripeptide was tested for its *in vitro* binding affinity to SMYD3. Then, the UniProt Peptide Search tool (https://www.uniprot.org/peptidesearch) was used to screen the whole human proteome to identify all proteins containing the P1–P19 tripeptides. Therefore, the 169671 human proteins reported in the UniProt/SwissProt database at the time of analysis (December 2018) were searched and mapped for each P-tripeptide, and 8650 proteins (termed P-proteins) were found to contain at least one P-tripeptide and thus considered potential SMYD3 interactors. In addition, a subset of 214 P-proteins showed at least 4 P-tripeptide occurrences [9], [10]. For each P-protein identified in this analysis, P-tripeptide matches and positions, gene names, protein names, lengths, functions, and Reactome IDs were recorded as annotated in the UniProt database.

### 2.2 *In silico* clustering of P-proteins in the ‘nonmutational epigenetic reprogramming’ cancer hallmark

This analysis was performed as previously described [10]. *In silico* clustering of the P-proteins involved in the ‘nonmutational epigenetic reprogramming’ cancer hallmark was performed by considering the relevant Reactome cluster (‘epigenetic regulation of gene expression’ pathway; Reactome Id: R-HSA-212165) *(https://reactome.org)* and the EpiFactors database (*http://epifactors.autosome.ru*) [32]. At the time of analysis (December 2022), the R-HSA-212165 Reactome pathway consisted of 156 human proteins, 61 of which were found to be P-proteins containing at least one P-tripeptide. In addition, among the 801 human proteins listed in the EpiFactors database, 348 tuned out to be P-proteins encompassing one or more P-tripeptides. After excluding redundant proteins between these two datasets, we obtained a final cluster of 361 P-proteins involved in the 'non-mutational epigenetic reprogramming' cancer hallmark. Among these 361 P-proteins, 35 were found to comprise at least 4 different P-tripeptides. Based on their oncogenic relevance and/or enrichment in P-tripeptides, selected candidates were further investigated for their ability to interact with SMYD3 *in cellulo*.

### 2.3 Analysis of SMYD3 interaction network using stringApp

Analysis of the updated SMYD3 interaction network was performed as previously described [10]. In a recent study, we generated an updated SMYD3 interaction network to incorporate 18 experimentally validated SMYD3 interactors known to have a role in cancer hallmark pathways [10]. Of these, ATM, BRCA2, CHEK2, MTOR, BLM, MET, p130, and AMPK (seven subunits) had been experimentally validated by our group [9], [10], while AKT1 [8], VGFR1[6], HSP90 [33], and RPB1 [5] had been validated by other research groups. To customize the SMYD3 interaction network with these proteins, we previously created a new Payload dataset using My Payload Plus, a plugin of the STRING database (https://version-11–0b.string-db.org) [10]. In the current report, we included EP300 and TRRAP in the Payload dataset described above to obtain a further updated SMYD3 interaction network. The resulting list of experimentally validated SMYD3 interactors (EP300, TRRAP, ATM, BRCA2, CHEK2, MTOR, BLM, MET, p130, 7 AMPK subunits, in addition to AKT1, VGFR1, HSP90, and RPB1) was used to search against the STRING database to evaluate all functional interactions linking these SMYD3 partners. Network analysis was performed at medium stringency (STRING score = 0.4). According to the default criteria of the STRING database, proteins were linked based on neighborhood, gene fusion, co-occurrence, co-expression, experimental evidence, existing databases, and text mining, with solid lines representing the functional links between proteins (nodes) and their thickness being proportional to the confidence level of the association. Next, we imported these STRING data into the Cytoscape server using StringApp 1.2 (Cytoscape app store, http://apps.cytoscape.org/apps/stringapp) [34]. StringApp has the advantage of including STRING and Cytoscape resources in the same workflow. Moreover, it allows to easily import STRING networks into Cytoscape, while keeping the appearance and many of the STRING features, and combines data from linked databases. In agreement with StringApp default criteria, interacting proteins were connected based only on experimental evidence.

### 2.4 Cell cultures

The HGC-27, HCT-116, and HT-29 cell lines were purchased from ATCC. The HGC-27 cell line was established by culture of a metastatic lymph node from a patient with gastric cancer diagnosed histologically as undifferentiated carcinoma [35]. HCT-116 cells are human colorectal carcinoma cells derived from an adult male [36]. Importantly, they harbor a C-terminal truncating mutation in EP300 [37]. HT-29 cells are human colorectal adenocarcinoma cells isolated from a primary tumor of an adult female patient [38]. All these cell lines are adherent with an epithelial morphology and were cultured in DMEM (#11360–070, Gibco) with 10% FBS (#0270–106, Gibco) and 100 IU/ml penicillin-streptomycin (#15140–122, Gibco). Cell cultures were performed under standard conditions in a 37 °C and 5% CO_2_ incubator and were routinely tested to be mycoplasma-free (#117048, Minerva). Cell lines were maintained in the exponential phase according to ATCC guidelines.

### 2.5 Co-immunoprecipitation

Cells (4 ×10^6^ cells/100 mm dish) were collected and homogenized in lysis buffer (50 mM Tris-HCl pH 7.4, 5 mM EDTA, 250 mM NaCl, and 1% Triton X-100) supplemented with protease and phosphatase inhibitors. The coupling phase between Dynabeads Protein A (#10001D, ThermoFisher Scientific) and the selected primary antibodies, i.e., anti-SMYD3 (#12859 Cell Signaling Technologies), anti-p300 (#57625 Cell Signaling Technologies), anti-TRRAP (#3967 Cell Signaling Technologies), anti-GFP (#2956 Cell Signaling Technologies) as an unrelated protein control, and anti-IgG (#2729 Cell Signaling Technologies) as a negative control, was performed in T-PBS (PBS+0.01% TWEEN) for 40 min at room temperature on a rocking platform. Then, samples were incubated with antibody-Dynabeads complexes for 3 h at room temperature on a rocking platform and immunoprecipitated. Immunoprecipitated proteins were extensively washed with lysis buffer, resuspended in Laemmli buffer, separated on a polyacrylamide gel, transferred to nitrocellulose membranes, and then subjected to immunoblot analyses. Immunoblot analyses were performed using anti-SMYD3 (#12859 Cell Signaling Technologies), anti-p300 (#57625 Cell Signaling Technologies), and anti-TRRAP (#3967, Cell Signaling Technologies). After incubation with rabbit IgG HRP (#NA934V, GE Healthcare), the signal was revealed using the ECL-plus chemiluminescence reagent (GE Healthcare) according to the manufacturer’s instructions. Input corresponds to 10% of the whole cell lysate.

## 3 Results

### 3.1 *In silico* clustering of P-proteins with epigenetic functions and analysis of P-tripeptide distribution

Recently, we performed a comprehensive *in silico* analysis of all human protein sequences encompassing a series of rare tripeptides, termed P1-P19, to gain insight into novel SMYD3 oncogenic functions [9], [10]. These tripeptides were first assessed for their *in vitro* binding affinity to SMYD3 and then used as *in silico* probes to search the whole human proteome for putative SMYD3-interacting proteins [9]. We found 8650 proteins containing at least one P-tripeptide (termed P-proteins), among which we identified various SMYD3 interactors, including ATM, BRCA2, CHK2, MTOR, BLM, MET, p130, and AMPK [9], [10]. These interactors are well-known major effectors of at least one cancer hallmark [10], [11].

**Fig. 1:**
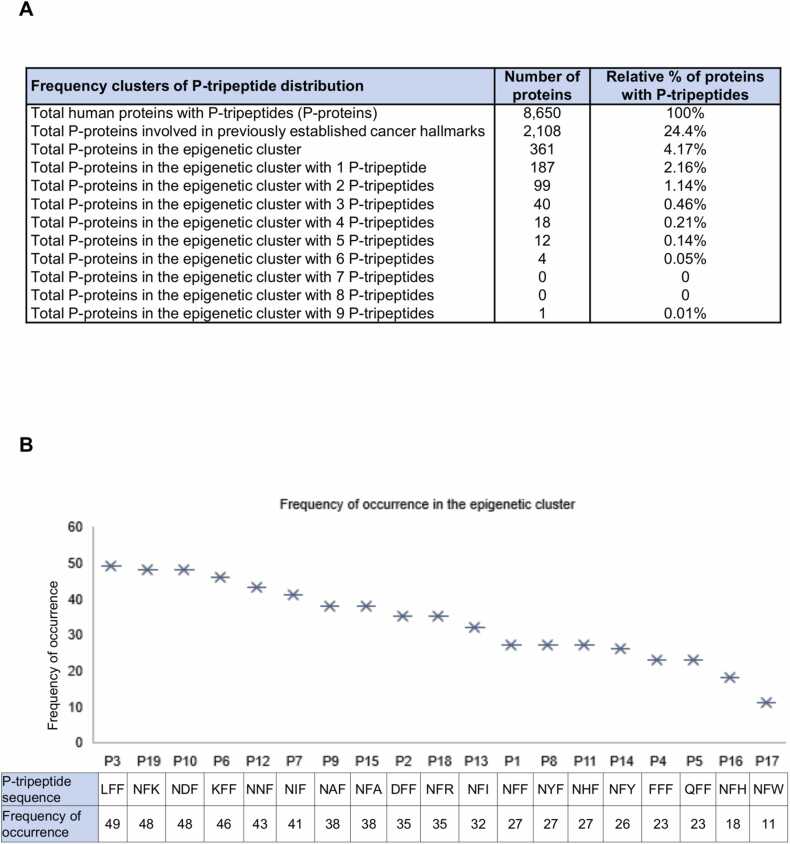
Quantitative analysis of P-tripeptide distribution in proteins involved in the emerging ‘nonmutational epigenetic reprogramming’ cancer hallmark. (A) Clustering of P-proteins involved in the ‘nonmutational epigenetic reprogramming’ cancer hallmark based on the frequency of total P-tripeptide occurrences. (B) Frequency graph of the occurrence of each P-tripeptide in the ‘nonmutational epigenetic reprogramming’ protein cluster.

In the current study, starting from this set of 8650 P-proteins, we identified 361 SMYD3 interactor candidates clustered as having epigenetic-related functions based on their annotation in the corresponding UniProt entry and on the pertinent Reactome pathway and EpiFactors databases at the time of analysis (December 2022) (Fig. 1A, Appendix Table S1). In particular, as part of this *in silico* clustering, we examined all the 117 proteins annotated in the ‘epigenetic regulation of gene expression’ Reactome cluster (Reactome Id: R-HSA-212165), 45 of which were found to contain P-tripeptides. To broaden our analysis, we also searched the EpiFactors database, which provides information about epigenetic regulators. In total, we obtained a final set of 361 P-proteins involved in pathways related to the emerging ‘nonmutational epigenetic reprogramming’ cancer hallmark.

Then, we performed a quantitative analysis of P-tripeptide distribution in these 361 P-proteins. To this end, we sub-clustered them based on the frequency of P-tripeptide occurrences (Fig. 1A). As expected, the number of proteins within each P-tripeptide frequency cluster decreases with the increasing number of P-tripeptide matches. This analysis also showed that the distribution of our P-tripeptides depends on the number of codons by which they are encoded (Fig. 1B, Appendix Table S1), in agreement with the theory of rare amino acids and as previously observed [10], [39], [40], [41]. In particular, we found that the less frequent tripeptide, i.e., P17 (NFW), comprises tryptophan, which is encoded by only one codon, while the most frequent tripeptide, i.e., P3 (LFF), contains leucine, which is encoded by six different codons (Fig. 1B).

**Fig. 2:**
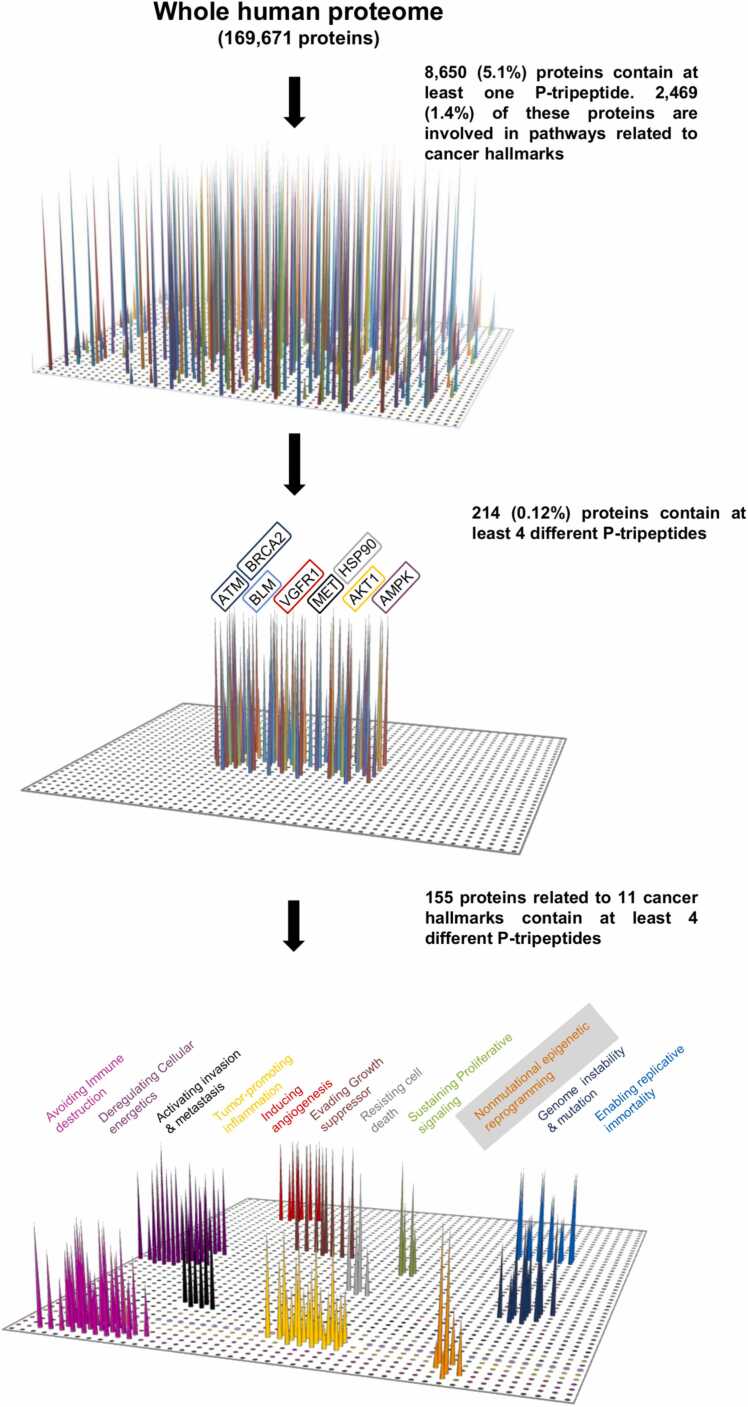
Procedural scheme of the qualitative analysis of P-tripeptide distribution in proteins involved in cancer hallmarks. Distribution of each P-tripeptide in all proteins annotated in the UniProt/SwissProt database (analysis performed in December 2018, https://www.uniprot.org). The human proteome was screened to search for exact matches of each P-tripeptide. Among the 8650 P-proteins identified, 2469 are involved in pathways related to the emerging ‘nonmutational epigenetic reprogramming’ (361) [11] or the ten previously established (2108) cancer hallmarks, and 214 contain at least four different P-tripeptides. In this subset, 155 proteins are included in clusters related to these 11 cancer hallmarks. Proteins were clustered based on their biological function as annotated in the corresponding Uniprot entry, and the clustering was confirmed in the Reactome (*https://reactome.org*) and EpiFactors (*http://epifactors.autosome.ru*) databases.

Subsequently, we performed a qualitative analysis to identify the SMYD3 interactor candidates that were most relevant for their epigenetic role in cancer processes. To this end, we evaluated the 361 P-proteins involved in the ‘nonmutational epigenetic reprogramming’ cancer hallmark for their enrichment in P-tripeptides. Intriguingly, we found that 35 proteins within this cluster were significantly enriched in P-tripeptides (at least 4 P-tripeptide matches), and 25 comprised at least 4 different P-tripeptides (Appendix Table S1). Overall, the total number of P-proteins involved in pathways related to the emerging ‘nonmutational epigenetic reprogramming’ (361) or the ten previously established (2108) [10] cancer hallmarks was 2469 (Figs. 1A, 2). Notably, a total of 155 P-proteins comprising at least 4 distinct P-tripeptides were included in clusters related to these 11 hallmarks of cancer (Fig. 2) [10].

### 3.2 EP300 and TRRAP as novel SMYD3 interactors involved in the ‘nonmutational epigenetic reprogramming’ cancer hallmark

To gain further insight into the role of SMYD3 in cancer-related epigenetic processes, we investigated in depth the interaction between SMYD3 and specific P-proteins involved in the emerging ‘nonmutational epigenetic reprogramming’ cancer hallmark (Fig. 2). This approach is validated by the evidence that various established SMYD3 interactors, such as VGFR1, AKT1, HS90A, MET, BLM, AMPK, ATM, and BRCA2, show a significant enrichment in P-tripeptides (Fig. 2) [1], [10]. Based on this *in silico* methodology, we identified EP300 and TRRAP as potential novel SMYD3-interacting proteins. These P-tripeptide-containing putative SMYD3 interactors were selected based on their prominent role in epigenetic regulation. Indeed, EP300 and TRRAP are components of large multisubunit HAT complexes that regulate the transcription of major oncogenes via chromatin remodeling [13], [26].

**Fig. 3:**
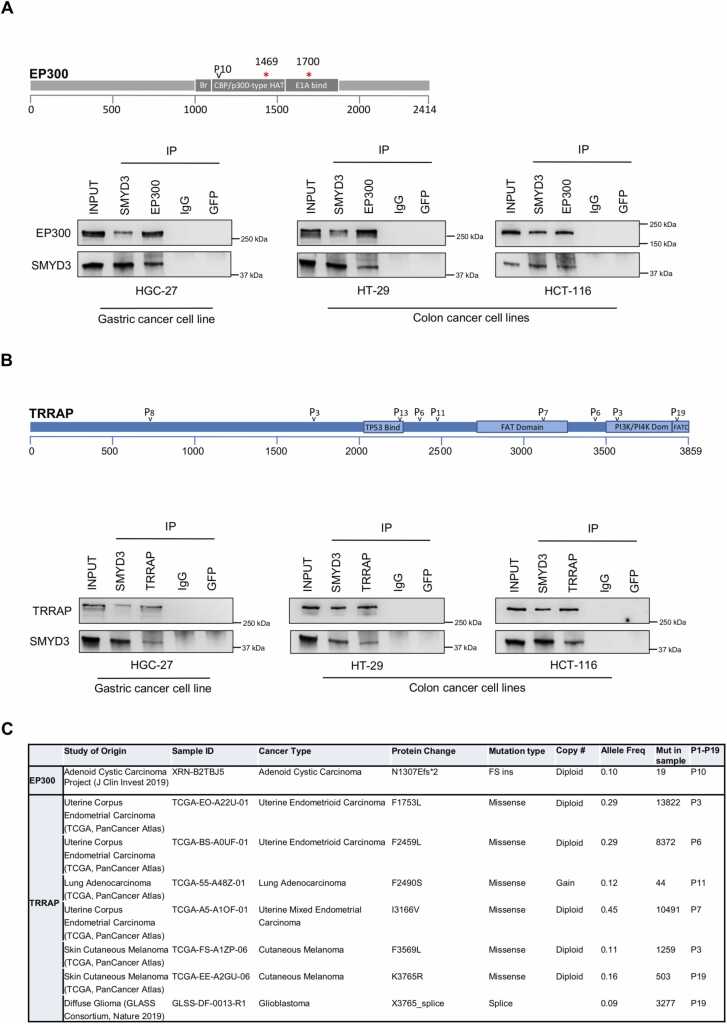
*In cellulo* validation of the SMYD3 interactions identified *in silico*. (A) Upper panel: P-tripeptide localization in the CBP/P300-type HAT domain of EP300. The asterisks indicate the truncating mutations found in HCT-116 cells. Lower panel: Co-immunoprecipitation of endogenous SMYD3 and EP300 in HGC-27 gastric cancer and HT-29 and HCT-116 colon cancer cell lines. (B) Upper panel: P-tripeptide localization in specific domains of TRRAP. Lower panel: Co-immunoprecipitation of endogenous SMYD3 and TRRAP in HGC-27 gastric cancer and HT-29 and HCT-116 colon cancer cell lines. Input corresponds to 10% of the whole cell lysate. Anti-IgG was used as a matched isotype negative control, and anti-GFP was used as an unrelated protein control. The presented results are representative of at least three independent experiments. Uncropped images of the immunoblots are shown in supplementary figure 1. (C) Somatic genetic alterations involving the localization of the P-tripeptides mapping to TRRAP and EP300 as reported in all curated and non-redundant cancer datasets available in the cBioPortal database (https://www.cbioportal.org/).

Based on our *in silico* analysis, EP300 contains one P-tripeptide (P10 at amino acid (aa) position 1307) located in the CBP/P300-type HAT domain, suggesting that this domain may be involved in SMYD3-EP300 interaction (Fig. 3 A, upper panel). To validate this interaction *in cellulo*, we performed co-immunoprecipitation assays in gastrointestinal cancer cell lines (HGC-27, HT-29, and HCT-116). Importantly, HCT-116 cells harbor two different frameshift mutations (c.4408del and c.5099del) in the EP300 gene, which result in truncated protein products lacking the C-terminal region [37]. Immunoprecipitation of whole cell lysates with an antiserum against SMYD3 or EP300, followed by immunoblotting, revealed that SMYD3 interacts with EP300 in all cell lines tested, including HCT-116, indicating that this interaction likely occurs at the N-terminal region of EP300 (Fig. 3 A, lower panel). This hypothesis is further supported by the fact that P10 is also located in this region.

TRRAP contains seven different P-tripeptides, i.e., P3 at aa positions 1752 and 3567, P6 at aa positions 2457 and 3457, P7 at aa position 3165, P8 at aa position 755, P11 at aa position 2488, P13 at aa position 2345, and P19 at aa position 3763 (Fig. 3B, upper panel). Interestingly, some of these P-tripeptides are located in the FAT and FATC domains and in the TP53 and PI3K-PI4K interaction domains, suggesting that these regions may also mediate the interaction with SMYD3. The physical interaction between endogenous SMYD3 and TRRAP was validated by co-immunoprecipitation assays in the HGC-27, HT-29, and HCT-116 gastrointestinal cancer cell lines. Our results revealed that SMYD3 is a molecular partner of TRRAP in these cells (Fig. 3B, lower panel).

The biological relevance of the EP300 and TRRAP sites encompassing P-tripeptides is supported by the evidence that some of them are involved in genomic alterations reported in cancer patient-derived datasets on the cBioportal website (Fig. 3 C). In particular, we found that the site of the P10 tripeptide in EP300 sequence is involved in an EP300 truncating mutation, N1307Efs* 2, detected in adenoid cystic carcinoma tumors (Fig. 3 C). Moreover, the sites of the P3, P6, P7, P11, and P19 tripeptides in TRRAP sequence are the targets of various reported TRRAP missense and splice mutations found in tumors of different origin (Fig. 3 C).

While the data obtained *in cellulo* confirm the technical suitability of our *in silico* methodology [10], further studies are needed to better characterize the crosstalk between SMYD3 and EP300/TRRAP and gain insight into the functional mechanisms underlying these novel SMYD3 interactions in epigenetic processes. For example, it can be speculated that EP300, TRRAP, and SMYD3 may contribute to the activation of a critical oncogene such as E1A. Notably, the E1A-binding proteins pRb, p107, and p130, as well as cAMP, CBP/P300, p400, and TRRAP have been implicated in oncogenic transformation [42].

**Fig. 4:**
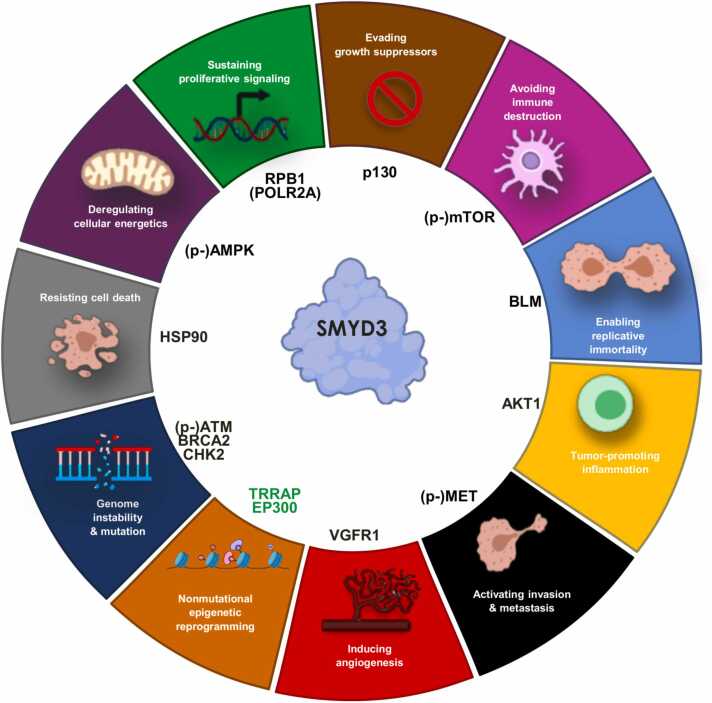
SMYD3 molecular interactors involved in cancer hallmarks. Diagram of selected SMYD3 interactors involved in pathways related to the emerging ‘nonmutational epigenetic reprogramming’ and the ten previously established cancer hallmarks. Previously validated SMYD3 interactors are shown in black, while the novel SMYD3 interactors identified in this study are shown in green.

**Fig. 5:**
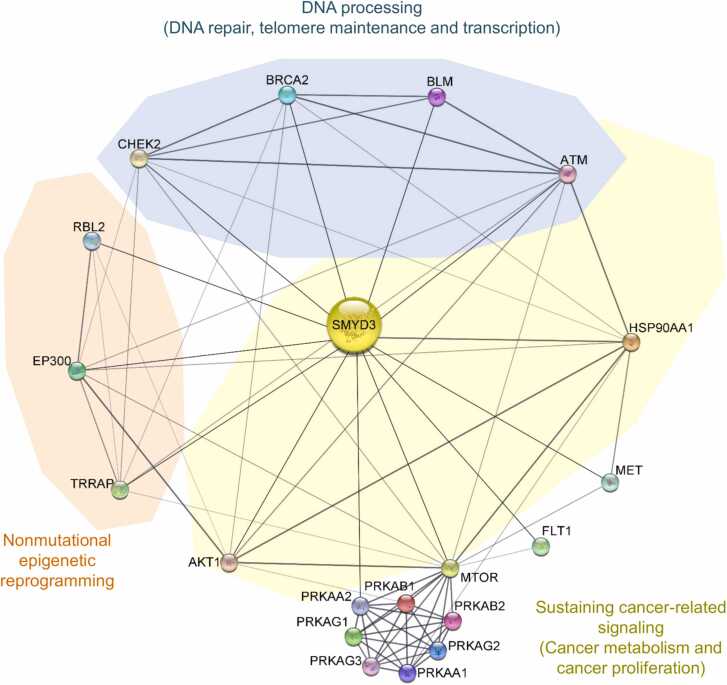
Updated SMYD3 interaction network. The updated SMYD3 interaction network was created using the My Payload Plus plugin for the STRING database to customize a Payload dataset of experimentally validated SMYD3 interactors involved in cancer hallmark processes. Nodes and edges of SMYD3 functional associations are represented based on STRING database criteria (https://string-db.org) and visualized with stringApp (Cytoscape app store, http://apps.cytoscape.org/apps/stringapp). Solid lines represent the functional linkages between proteins (nodes) and their thickness is proportional to the confidence level of the association. The protein name of each interactor is indicated in agreement with the UniProt nomenclature (https://www.uniprot.org/).

EP300, TRRAP, and other previously identified SMYD3 interactors are summarized in Fig. 4, together with the cancer hallmarks in which they are involved [10]. This schematic diagram provides a general overview of the complexity of SMYD3 functions in the framework of cancer-associated processes. These functions are mediated by an intricate network of interactions. Thus, based on the above-described *in silico*, *in cellulo*, and patient-derived molecular findings, we used stringApp 1.2 to create an updated SMYD3 interaction network including TRRAP and EP300 as novel experimentally validated SMYD3 interactors (Fig. 5) [34].

Overall, these data suggest that SMYD3 orchestrates epigenetic, metabolic, and proliferative events in the adaptive response of tumor cells during carcinogenesis and cancer progression, acting as a crucial modulator of major oncogenic processes, such as epigenetic regulation, DNA processing, and cancer-related signaling (Fig. 5). In this scenario, the elucidation of the multifaceted role of SMYD3 in cancer may lead to new and more effective therapeutic approaches.

## Funding

This research was funded by the Italian 10.13039/100009647Ministry of Health “Ricerca Corrente 2021–2023” to C.S.; “Ricerca Corrente 2022–2024” to C.F.; “Ricerca Corrente 2022–2024” to V.D.; “Ricerca Corrente 2023–2025” to V.G.; AIRC IG-23794 2020–2024 to C.S.; the ‘Starting Grant’ SG-2019–12371540 to P.S., and an 10.13039/501100005010AIRC Fellowship for Italy to M.L.S. (ID26678–2021).

## CRediT authorship contribution statement

**F.C**: conceptualization, methodology, software, validation, investigation, data curation, writing – original draft, writing – review & editing, visualization. **M.L.S.**: methodology, validation, investigation, data Curation. **E.D.N.**: investigation, data curation. **A.P.**: investigation, data curation. **G.F.**: investigation, data curation. **K.D.M.**: investigation, data curation. **P.S.**: visualization, data curation. **V.D.**: visualization, data curation. **V.G.**: review & editing, supervision. **C.S.**: conceptualization, supervision, project administration.

## Declaration of Competing Interest

The authors whose names are listed immediately below certify that we have NO affiliations with or involvement in any organization or entity with any financial interest (such as honoraria; educational grants; participation in speakers’ bureaus; membership, employment, consultancies, stock ownership, or other equity interest; and expert testimony or patent-licensing arrangements), or non-financial interest (such as personal or professional relationships, affiliations, knowledge or beliefs) in the subject matter or materials discussed in this manuscript.

## Appendix A Supplementary material

.

Supplementary material Fig. 1**: Uncropped images of the immunoblots shown in Fig. 3 A-B.**
