# Supplementary figures and images for: The chromatin remodeling factors EP300 and TRRAP are novel SMYD3 interactors involved in the emerging ‘nonmutational epigenetic reprogramming’ cancer hallmark
Source: Comput Struct Biotechnol J. 2023 Oct 12;21:5240–8. doi: 10.1016/j.csbj.2023.10.015 (PMC10632561; doi:10.1016/j.csbj.2023.10.015)

A

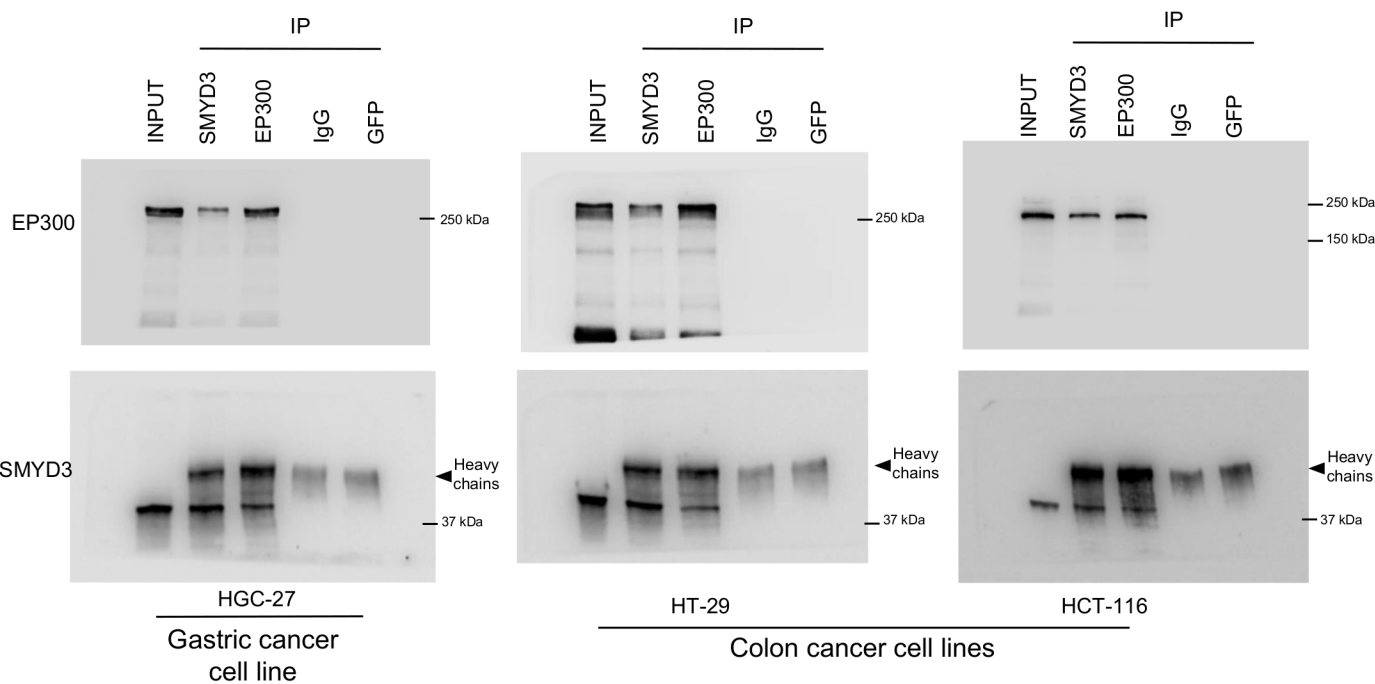

B

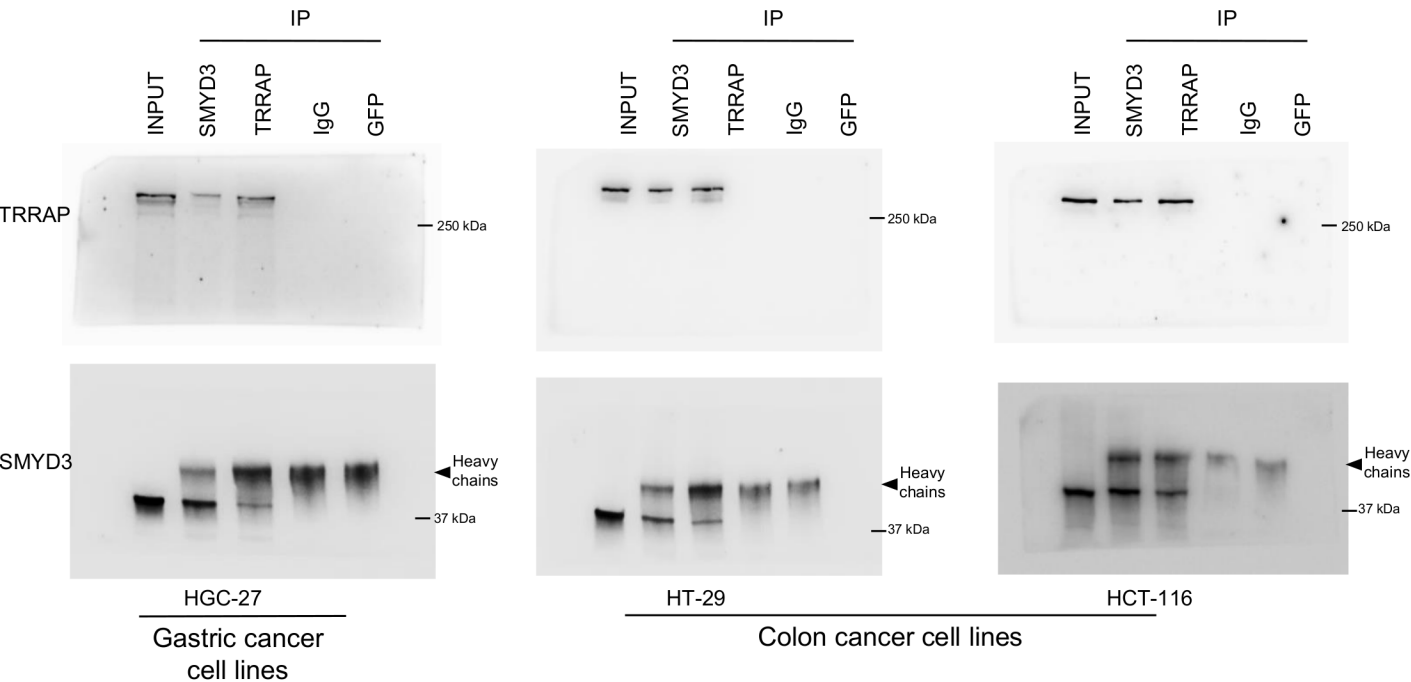

Supplement: Supplementary file 2 — Supplementary material Fig. 1: Uncropped images of the immunoblots shown in Fig. 3 A-B. [file mmc2.pdf]
